# Making Quality Improvement Data Meaningful for Long-Term Care Administrators

**DOI:** 10.1093/geroni/igaa057.590

**Published:** 2020-12-16

**Authors:** Lisa Cranley, Lori Weeks, T K T (Thomas) Lo, Peter Norton, Carole Estabrooks

**Affiliations:** 1 University of Toronto, Toronto, Ontario, Canada; 2 Dalhousie University, Halifax, Nova Scotia, Canada; 3 University of Alberta, Edmonton, Alberta, Canada; 4 University of Calgary, Calgary, Alberta, Canada

## Abstract

Tailoring feedback data to engage end-user stakeholders when sharing organizational context data is a central component of quality improvement and integrated knowledge translation. For over a decade, our research team has collected survey data (using the validated Alberta Context Tool) on modifiable aspects of organizational context from long-term care (LTC) staff (e.g., nurses, unregulated providers) across a representative cohort of 94 LTC facilities in Western Canada. We have fed back data at the facility and care unit level with the goal of making research findings more useful for decision-making and aiding improvement efforts. We have used a binary method (more favourable / less favourable organizational context) to report multidimensional data. While useful to our stakeholders (e.g., administrators) we are continually seeking ways to increase the detail in our reporting, while maintaining usability for stakeholders. We have now developed a more detailed method – the context rank summary, which displays rankings of care units within and across LTC facilities. In this study, we used a qualitative descriptive design to explore perspectives of administrators and managers (leaders) from LTC facilities on the two different methods for reporting survey data. We conducted a total of three focus groups with 16 leaders in the Maritimes and Ontario, Canada. Transcripts were analysed using content analysis. Leaders preferred a feedback report that combines a binary method with the greater detail of the context rank summary. Providing organizational context data that is more meaningful, relevant and actionable could offer an additional path to identifying areas for improvement.

